# Behavioral drivers of AI nursing acceptance in the Greater Bay Area: a family-caregiver perspective on trust and risk

**DOI:** 10.3389/fpubh.2025.1650804

**Published:** 2025-10-02

**Authors:** Chenxi Ye, Zeyu Wang, Miaohui Wu, Runhao Kang, Fujiang Yuan, Chen Chen

**Affiliations:** ^1^School of Art and Design, Guangdong University of Technology, Guangzhou, China; ^2^School of Literature and Media, Nanfang College Guangzhou, Guangzhou, China; ^3^Institute for Design, Hebei Academy of Fine Arts, Shijiazhuang, China; ^4^School of Computer Science and Technology, Taiyuan Normal University, Taiyuan, China

**Keywords:** AI nursing technologies, family caregivers, trust, perceived risk, UTAUT

## Abstract

The rapid aging of the population in the Guangdong-Hong Kong-Macao Greater Bay Area (GBA) has increased demand for smart healthcare solutions. Artificial intelligence (AI)-based nursing technologies show promise in alleviating care burdens, yet family caregivers—often the primary decision-makers—exhibit low adoption rates due to trust issues and risk perception. This study investigated factors influencing caregivers’ behavioral intention to adopt AI nursing technologies by developing an extended Unified Theory of Acceptance and Use of Technology (UTAUT) model incorporating trust and perceived risk. A cross-sectional survey was conducted across hospitals and care institutions in the GBA (*n* = 163) and analyzed using Partial Least Squares Structural Equation Modeling (PLS-SEM). Results indicated that trust, perceived usefulness (performance expectancy), and institutional support (facilitating conditions) were positively associated with intention to adopt. Social influence also had a positive effect but was significantly weakened by perceived risk, while age moderated the effect of perceived difficulty on adoption intention. The findings highlight the importance of improving system transparency, tailoring interface design for older users, and building trust through institutional support, suggesting that policymakers and developers should prioritize inclusive, age-adaptive AI design and ethical governance to enhance caregiver acceptance and AI integration in older population.

## 1 Introduction

The global community is facing an unprecedented demographic challenge: rapid population aging. According to the Ministry of Civil Affairs and the National Working Commission on Aging of China, by the end of 2023, the number of individuals aged 60 and above in China had reached 296.97 million—21.1% of the total population—with those aged 65 and over accounting for 216.76 million (15.4%). Over the next decade, the country is projected to add more than 20 million older adults individuals annually, and the population of the oldest old (aged 80 and above) is expected to surpass 50 million by 2035 (1). This demographic shift, coupled with a rising burden of chronic disease, places immense strain on healthcare and caregiving systems.

In the economically vibrant and densely populated Guangdong–Hong Kong–Macao Greater Bay Area (GBA), population aging presents similarly urgent challenges. Ensuring access to high-quality and sustainable eldercare services has become a critical regional priority. According to a recent industry report by KPMG, the rapid advancement of artificial intelligence (AI) is reshaping opportunities in healthcare and eldercare. Over the coming decade, AI technologies are expected to drive transformative innovations—from AI-assisted diagnostic systems and personalized treatment plans to autonomous caregiving robots—potentially alleviating workforce shortages and enhancing service delivery (2).

The GBA has emerged as a testbed for AI-driven healthcare innovations. As reported by China Daily, public hospitals in Hong Kong are deploying AI systems to identify high-risk patients and enable early intervention. Locally developed models, such as the ophthalmic diagnostic system VisionFM, now demonstrate accuracy on par with or superior to expert clinicians. In Shenzhen, AI-assisted surgical planning platforms like “CARES Copilot” are being integrated into clinical practice. Collectively, these advancements signal a growing regional commitment to leveraging AI for more efficient, intelligent healthcare systems (3).

**Figure 1:**
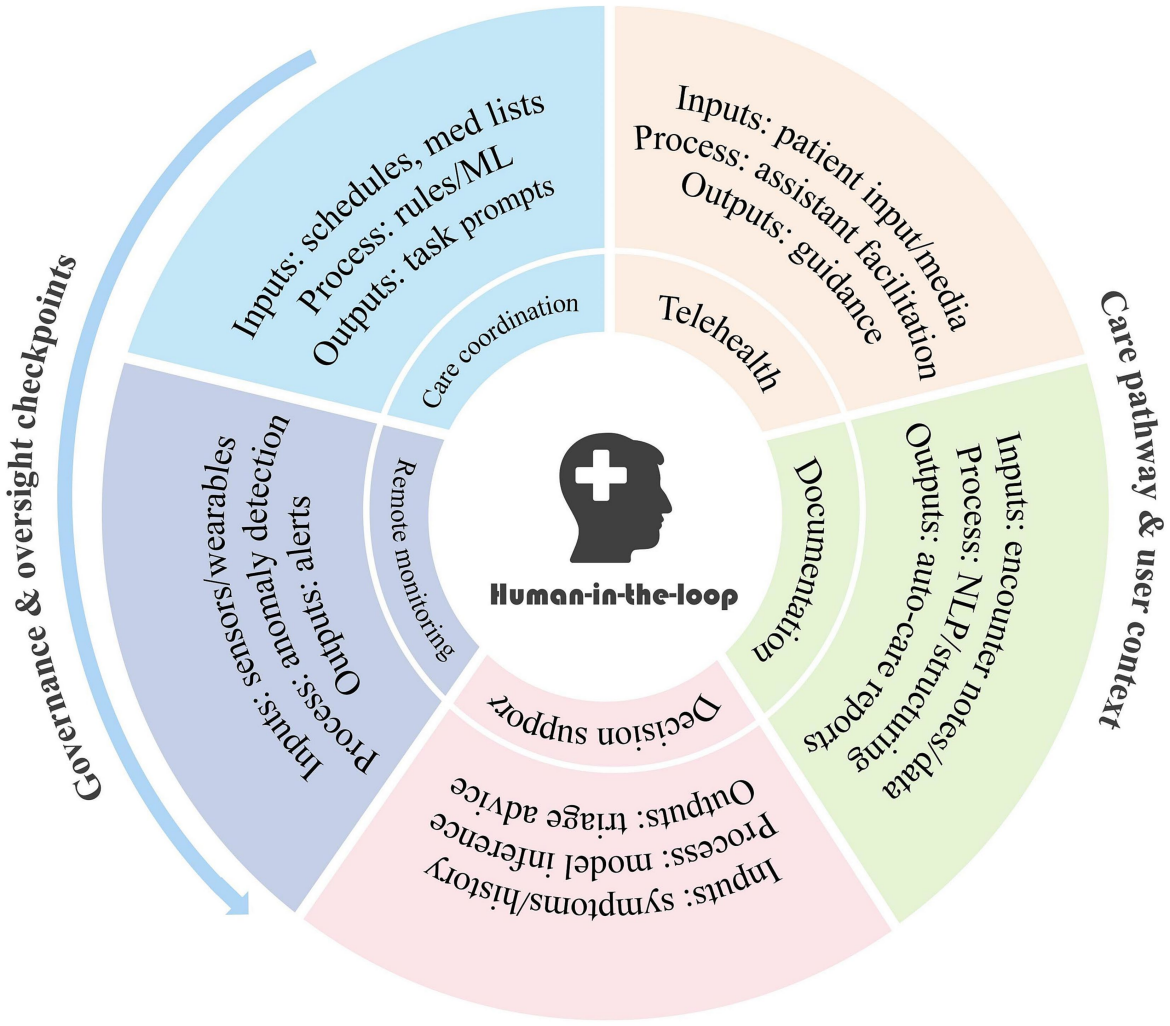
Applications of AI-driven healthcare systems in the Greater Bay Area (GBA).

To provide readers with a clearer overview of the diversity of AI-driven healthcare systems in the region, we have summarized the main categories of current applications in Figure 1, including remote monitoring, care coordination, telehealth, documentation, and decision support.

However, the realization of AI’s full potential hinges not only on technical capability, but also on end-user acceptance. In clinical and caregiving contexts—where safety, reliability, and ethics are paramount—trust plays a pivotal role. Despite AI’s promise, widespread skepticism persists among patients and their families. Studies indicate that 65.8% of surveyed adults express low trust in the use of AI in healthcare, and 57.7% doubt the healthcare system’s ability to prevent AI-related harm (4). Even as general health and AI literacy improve, trust deficits remain, suggesting that building acceptance will require deeper engagement and transparency (5).

Mistrust is often exacerbated by negative user experiences with overhyped or underperforming AI caregiving products. Such setbacks have made trust and perceived risk key barriers to adoption. Notably, when it comes to eldercare decisions, family members frequently serve as surrogate decision-makers. Many older adults rely on family due to cognitive limitations or unfamiliarity with digital tools, depending on them to install health apps, interpret electronic health records, or facilitate telehealth consultations (6, 7).

In this context, family caregivers often become de facto gatekeepers for AI adoption. If they harbor doubts or perceive high risk, they may withhold consent for AI-assisted care, regardless of its potential benefits (8). Therefore, understanding the perspectives of patients’ families—their willingness to adopt AI caregiving technologies and the factors shaping their trust—is crucial to unlocking the transformative value of AI in eldercare. As the GBA continues to accelerate AI integration into healthcare systems, addressing the needs, concerns, and behavioral intentions of these key stakeholders is essential for achieving meaningful, user-centered innovation.

## 2 Research questions

Although earlier research highlighted the persistent resistance to innovation in care services (9, 10), more recent evidence indicates that the COVID-19 pandemic has accelerated the adoption of digital health and also reshaped the dynamics of trust. For instance, Park et al.’s research found that the emotions of family caregivers strongly influence their willingness to adopt medical AI (6); After surveying 2,039 respondents, Nong and at found that although health literacy has improved, respondents’ trust in the use of AI in the healthcare system remains fragile (5). Similarly, a quantitative survey and analysis by Janne Kauttonen and other researchers on trust and acceptance of artificial intelligence applications in the healthcare sector revealed that doubts about AI-assisted healthcare persisted after the pandemic, mainly stemming from emotional and privacy concerns. These findings highlight the necessity of examining the acceptance of AI care in the post-pandemic care environment (11). In the context of sensor-based health monitoring, studies by T.F. Kummer have shown that hospitals in Germany and Australia deploying intelligent surveillance systems frequently encounter user anxiety triggered by a complex interplay of factors. These include uncertainty about the system’s effectiveness, concerns over privacy and security, and emotionally driven intuitive rejection (10). Such findings underscore that, in healthcare settings, technology adoption is shaped not only by rational cost–benefit evaluations but also by psychological and affective dimensions.

Located at the crossroads of Eastern and Western medical traditions and cultural frameworks, the Guangdong–Hong Kong–Macao Greater Bay Area (GBA) presents a distinctive context in which public perceptions of medical AI are shaped by a unique interplay of influences. The bases for trust and the ways users perceive risk in this region are likely to diverge significantly from those observed elsewhere. Nevertheless, existing research on public acceptance of AI-driven healthcare solutions within the GBA remains limited, with even fewer studies concentrating on the perspectives of family caregivers—a stakeholder group of considerable importance.

Conventional frameworks for studying technology acceptance, such as the Technology Acceptance Model (TAM) and the Unified Theory of Acceptance and Use of Technology (UTAUT), are commonly utilized to evaluate factors influencing the adoption of AI in healthcare and eldercare environments (12). Despite their broad application, these models frequently overlook essential psychological components, particularly trust and perceived risk (8). Although the UTAUT’s core elements—performance expectancy, effort expectancy, social influence, and facilitating conditions—offer explanatory power in general information systems research, their ability to predict user intentions diminishes in healthcare contexts, where the stakes of decision-making and potential errors are much higher.

In such settings, trust in AI’s reliability and safety, as well as perceived risks of unintended harm, become pivotal in shaping decisions. Overlooking these factors may obscure the reasons why some family members remain unwilling to authorize the use of AI-based care technologies, even when their potential benefits are clearly demonstrated (4, 8). Therefore, there is a compelling need to extend existing theoretical models by incorporating trust as a central antecedent and perceived risk as a moderating factor in explaining AI adoption behaviors in healthcare.

Based on these insights, this study seeks to answer the following core research question:

What factors influence family members’ willingness to adopt AI caregiving technologies in the Greater Bay Area, and how do trust and perceived risk shape this decision-making process?

To tackle this research gap, we construct an extended conceptual framework grounded in UTAUT, incorporating trust and perceived risk as pivotal psychological factors. Specifically, the model maintains the core UTAUT predictors—performance expectancy (perceived AI utility), effort expectancy (ease of use), social influence (social encouragement/pressure), and facilitating conditions (supportive resources/infrastructure)—as foundational determinants of behavioral intention. Trust is integrated as an additional direct antecedent, defined as family members’ assurance in the safety and competence of AI-assisted caregiving. Furthermore, we propose that perceived risk—conceptualized as the subjective assessment of potential adverse consequences linked to AI use—exerts a moderating effect on the trust-intention relationship. This synthesized framework seeks to provide a more holistic explanation of the psychological drivers shaping AI adoption choices in eldercare, particularly within the Greater Bay Area’s unique socio-technical environment.

## 3 Literature review

### 3.1 The current landscape of AI in nursing

Research on artificial intelligence (AI) in nursing and healthcare has flourished in recent years, characterized by multi-directional and multi-layered advancements. Functionally, AI has been employed across a wide spectrum of applications, including medication management, patient monitoring and engagement, and optimization of nursing administration (13, 14). A comprehensive systematic review identified core applications of AI in healthcare, such as diagnostic assistance, disease management, personalized health interventions, patient self-management, and improvements in hospital operations (15). These functionalities are increasingly being extended to nursing contexts.

In clinical practice, AI-driven decision support systems (DSS) can aid nurses in assessing patient conditions and formulating personalized care plans, thereby enhancing decision accuracy and reducing workload (16). In disease management and rehabilitation, intelligent monitoring devices and algorithms enable real-time tracking of vital signs and symptoms, allowing for timely alerts and interventions by healthcare providers or family caregivers—ultimately improving patient outcomes and self-management (17). AI technologies are also reshaping administrative workflows in nursing by streamlining staff scheduling, predicting bed turnover, and minimizing paperwork, thus improving overall service efficiency (18).

Several case studies highlight tangible progress in both domestic and international contexts. For example, deep learning models have demonstrated high accuracy in diagnosing hepatic diseases, offering a promising AI-assisted approach for future hepatology care (19). In inpatient monitoring, AI-enabled sensors provide continuous 24/7 surveillance and early risk detection, potentially reducing nurses’ burden and improving patient safety. However, some family members perceive such monitoring as intrusive, citing privacy concerns, emotional detachment, and skepticism toward technological reliability (20).

In caregiving and elder services, socially assistive robots and AI-enabled smart speakers have been introduced to provide companionship, medication reminders, and emergency alerts (20). Although pilot implementations show promise in enhancing patient well-being and safety, broader adoption remains constrained by emotional, ethical, and risk-related concerns (21).

Overall, AI in nursing represents a dual function of empowerment and efficiency enhancement—enabling care professionals to perform better and expanding the reach and responsiveness of nursing services. Buchanan et al. (22) argue that AI systems can alleviate administrative burdens, allowing nurses to focus on tasks that demand human empathy and clinical judgment. This human-AI collaboration model is widely regarded as the future of nursing.

Nevertheless, scholars urge caution, noting potential risks such as algorithmic bias, decision opacity, and system failures, which necessitate robust governance mechanisms to ensure the trustworthiness and reliability of AI in clinical practice (6, 23). The key principles identified for successful AI implementation in healthcare are trustworthiness, usability, and accessibility—AI must be perceived as reliable, easy to use, and readily available to patients and their families (24).

In the Guangdong–Hong Kong–Macao Greater Bay Area (GBA), AI nursing research exhibits unique characteristics. The region boasts strong investments in healthcare innovation, with notable projects from institutions such as the Hong Kong Hospital Authority (3). Robert (20) notes that the emergence of AI caregiving technologies is likely to reshape the healthcare workforce in the near future. For instance, Hong Kong hospitals have piloted AI assistants with cartoon avatars to remind patients to take medications, and have actively gathered feedback from patients and families (3). Similarly, smart older adults care programs in Guangdong report that trust from older adults and their families is critical to AI product adoption (25).

These regional experiences underline the interactive relationship between technological innovation and user acceptance. The success of AI in nursing is not solely determined by algorithmic sophistication or hardware capabilities; it also hinges on the system’s ability to address humanistic, ethical, and experiential expectations from users—especially patients and their families.

### 3.2 The UTAUT model and its applications in healthcare

In information systems, the Unified Theory of Acceptance and Use of Technology (UTAUT), introduced by Venkatesh et al. (26), is a widely utilized framework. It identifies four core determinants of behavioral intention toward technology adoption: Performance Expectancy (usefulness), Effort Expectancy (ease of use), Social Influence (peer/social pressure), and Facilitating Conditions (supportive environment). Empirical evidence demonstrates that these factors collectively predict up to 70% of intention variance, underscoring UTAUT’s status as a parsimonious and comprehensive model (26).

Since its introduction, UTAUT has been applied extensively across diverse sectors, including educational technology (27), healthcare (28), Emotional support (29), e-government (30), financial technologies (31), and mobile internet (32). These applications reaffirm the model’s utility in capturing the essential cognitive and contextual determinants of technology adoption.

Nevertheless, it is acknowledged that healthcare’s unique complexities may restrict the applicability of the standard UTAUT framework. Decisions in health contexts are often emotionally intense, carry high stakes, and encompass multifaceted psychological and ethical dimensions. Key influences such as trust, privacy worries, safety concerns, and moral judgments—not explicitly covered by core UTAUT—may play a decisive role in healthcare technology adoption (4, 8, 33).

As a result, numerous studies have proposed extensions to UTAUT by incorporating Trust and Perceived Risk as additional variables. For instance, Cao et al. (34) integrated trust, risk perception, and health consciousness into an extended UTAUT model to study mobile health app adoption among Japanese youth. Their findings showed that trust significantly boosted usage intentions, while perceived risk had a clear negative effect.

Thus, although UTAUT remains a powerful theoretical lens, it requires contextual adaptation to fully capture the nuances of technology acceptance in healthcare. This is particularly true in domains such as AI-enabled caregiving, where users—especially family members of patients—may base decisions more on emotional security and perceived risk than on functionality or convenience alone (18, 35).

Building upon the UTAUT framework, this study incorporates trust and perceived risk to create a model sensitive to the Greater Bay Area’s unique cultural and institutional context. The resulting hybrid model is designed to reveal patient families’ genuine perspectives and concerns about AI in caregiving, offering richer insights into the sociotechnical dynamics underlying adoption decisions.

## 4 Research hypotheses

Performance expectancy, as defined by Venkatesh et al. (26), denotes an individual’s perception that adopting a specific technology will enhance their task performance or goal attainment. Empirical evidence consistently links this expectancy to stronger intentions to adopt technologies (36). Conversely, effort expectancy captures the perceived ease of use and low effort required when utilizing a technology (26). This dimension similarly predicts adoption intention (37). To enhance clarity for nursing participants, effort expectancy was assessed using reverse-scored items in this study.

Social influence is conceptualized as an individual’s belief about whether significant others (e.g., society, hospitals, physicians) expect them to use a particular technology (26). Within AI-based nursing, this translates to perceptions of AI acceptance by these key stakeholders. Facilitating conditions represent the perceived availability of resources and support enabling effective technology use (26).

Building on UTAUT’s theoretical foundation, the following hypotheses are advanced:

H0: Family caregivers' wilingness to accept AI-based nursing significantly influences their actual usage behavior.

H1: Performance expectancy has a significant positive effect on behavioral intention to use AI-based nursing.

H2: Reverse-coded effort expectancy has a significant negative effect on behavioral intention to use AI-based nursing.

H3: Social influence has a significant positive effect on behavioral intention to use AI-based nursing.

H4: Facilitating conditions have a significant positive effect on behavioral intention to use AI-based nursing.

Considering the specific context of AI in nursing, this study further proposes that trust in AI shapes nurses’ acceptance willingness (38). Therefore, an additional hypothesis is formulated:

H5: Trust in AI has a significant positive effect on family caregivers’ behavioral intention to use AI-based nursing.

As AI-based nursing is an emerging technology, nurses’ decision-making may involve more complex psychological and organizational factors. For instance, under high perceived risk, nurses may question whether AI can make accurate diagnoses; perceived risk may suppress social normative pressure, rendering social influence less effective; and there may be cognitive dissonance between perceived risk and trust (e.g., concerns about algorithmic transparency). Therefore, perceived risk is introduced as a moderating variable:

H6a: Perceived risk may weaken the positive effect of performance expectancy on behavioral intention.

H6b: Perceived risk may weaken the positive effect of social influence on behavioral intention.

H6c: Perceived risk may weaken the effect of trust in AI on behavioral intention.

Age is also introduced as a moderating variable. Older individuals tend to experience a decline in working memory capacity, making them more cognitively burdened by multi-step AI operations and more susceptible to frustration (39). Furthermore, older users are more emotionally motivated and less susceptible to social approval or authoritative opinions (40). Glikson and Woolley (38) also suggest that a high level of trust can reduce perceived risk in older adults. Based on this, the following hypotheses are proposed:

H7a: Older caregivers are more sensitive to the perceived difficulty of operating AI systems.

H7b: Age weakens the effect of social influence on behavioral intention.

H7c: Increased age strengthens the effect of trust in AI on behavioral intention.

**Figure 2:**
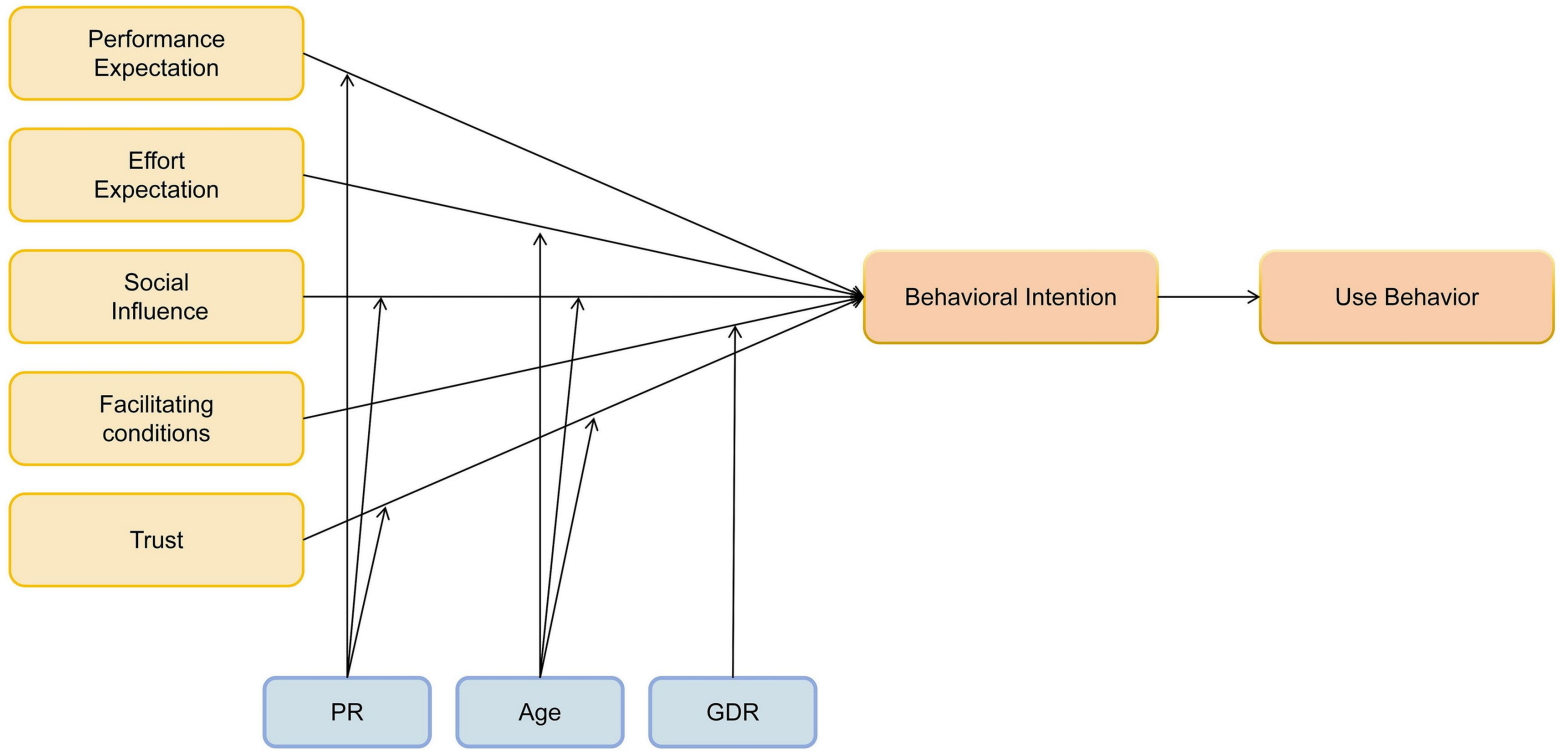
Research model.

Venkatesh and Morris (26) found that gender moderates the effect of facilitating conditions on technology acceptance, with women being more influenced by the availability of support. Additionally, research in healthcare settings has shown that female nurses tend to have greater needs for team-based resources and collaboration. Therefore, the following hypothesis is proposed (Figure 2):

H8: Sensitivity to facilitating conditions differs significantly between genders.

## 5 Research methodology

### 5.1 Measures and operational definitions

All constructs in this study were measured using reflective multi-item scales adapted from established literature to ensure validity and reliability. The items were rated on a five-point Likert scale ranging from 1 (strongly disagree) to 5 (strongly agree). To enhance comprehension among family caregivers—who may be unfamiliar with specific AI technologies—we provided a clear explanation in the survey introduction: “AI nursing technologies refer to intelligent systems or devices that assist in older adults care, such as health monitoring sensors, medication reminders, emergency alert systems, or companion robots.” Detailed operational definitions and measurement items for each construct are provided in the Table 1.

**Table 1:** Operational definitions and measurement items.

Constructs	Operational definition	Questions
Performance Expectation	The extent to which caregivers believe that using AI nursing technology improves the efficiency and quality of caregiving.	I believe that AI-assisted nursing diagnosis and treatment can enhance the efficiency of care.
		AI technology helps improve the quality of patients’ rehabilitation.
		Using an AI-assisted system enables me to better understand the patient’s condition.
Effort Expectation	The degree of ease associated with learning and using AI nursing technology.	It’s not easy for me to learn to use AI-assisted systems.
		I think using an AI system requires too much technical knowledge.
		I cannot quickly adapt to the operation process of AI-assisted care.
Social Influence	The extent to which caregivers perceive that important others expect or encourage them to use AI nursing technology.	Important others (such as doctors and nurses) think that I should use an AI-assisted system.
		The recognition of AI in healthcare in society has influenced my acceptance of it.
		The usage trend in hospitals will influence my view on AI systems.
Facilitating conditions	The perceived availability of resources and institutional support to enable the use of AI nursing technology.	I think hospitals have the resources (such as equipment and networks) to use AI systems.
		If I encounter any problems, there is technical support available to assist me in solving them.
		Medical institutions have provided sufficient AI-related training or explanations for family members.
AI Trust	The confidence of caregivers in the reliability, safety, and fairness of AI nursing systems.	I believe that the AI system will make reasonable nursing judgments.
		I believe that AI technology can safeguard the basic rights of patients.
		I trust that the AI system deployed by the hospital is reliable.
Perceived Risk	The subjective expectation of potential losses or adverse consequences associated with using AI nursing technology.	I’m worried that the AI system might malfunction and affect patient safety.
		I’m worried that using AI might leak patients’ private information.
		I’m uneasy about whether the AI’s judgment is accurate.
Behavioral Intention	The extent to which caregivers intend to use or recommend AI nursing technology.	If conditions permit, I am willing to use AI-assisted care services.
		In the future, I will give priority to medical institutions that incorporate AI systems.
		I am willing to recommend AI-assisted care services to others.
UB	The actual or self-reported use of AI nursing technology in caregiving practice.	If the hospital continues to offer AI-assisted care services, I will choose to keep using them.
		In the future nursing process, I hope to continue to rely on AI systems to assist in decision-making.
		Even without a doctor’s advice, I am willing to actively use AI-assisted functions.
		I have already incorporated AI-assisted care services as part of my medical care.
		I tend to continue using AI systems in other similar medical facilities.

Control variables such as age (continuous) and gender (categorical) were also included.

### 5.2 Sample recovery and analysis

**Figure 3:**
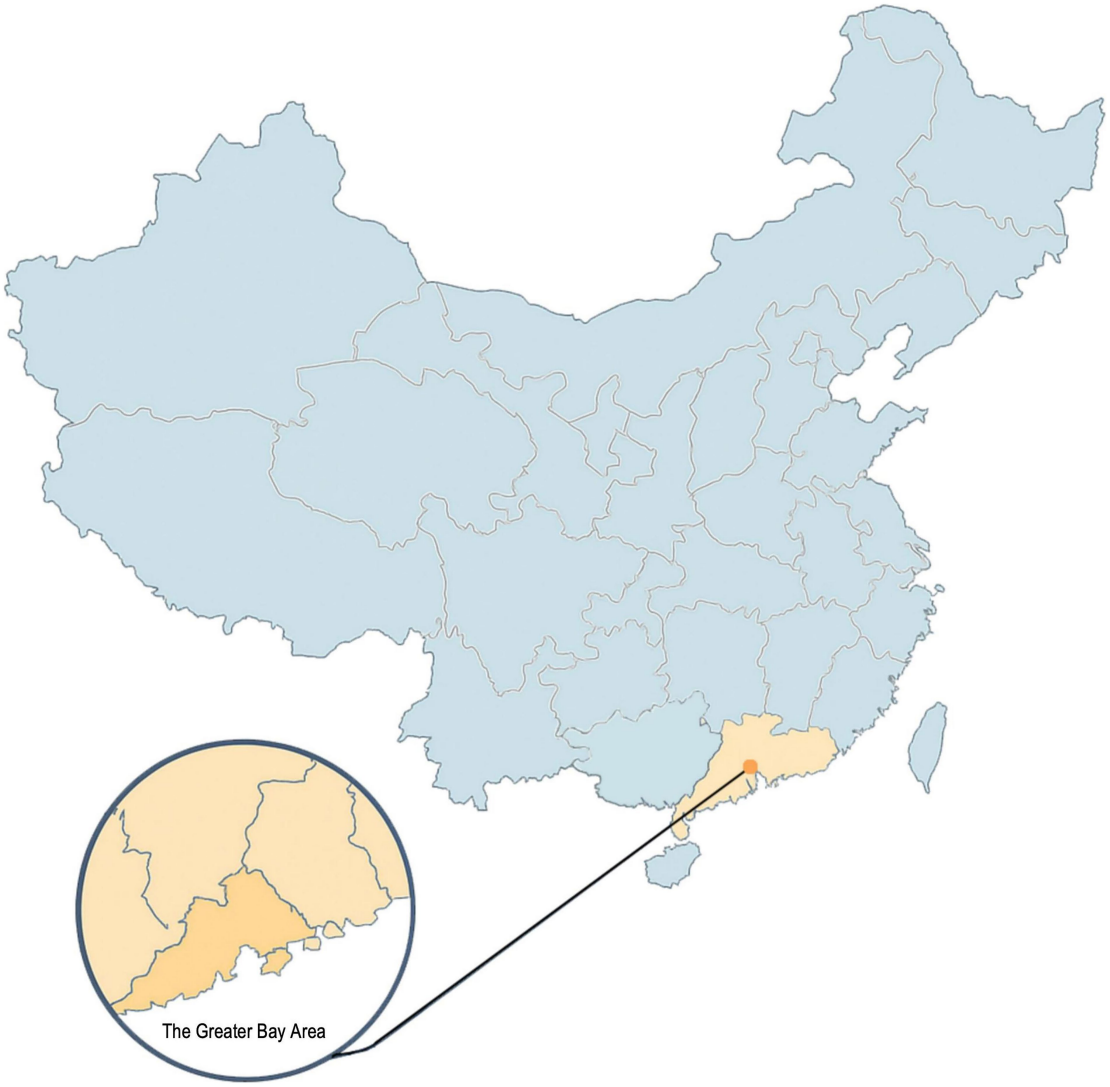
The location of the Pearl River Delta.

This study targeted family members responsible for medical decision-making or participating in caregiving processes for older adults patients within the Guangdong-Hong Kong-Macao Greater Bay Area, encompassing nine cities in central and southern Guangdong Province, Hong Kong Special Administrative Region, and Macao Special Administrative Region (Figure 3).

Recruitment was conducted through both online and offline channels. For the online sample questionnaires were distributed via academic networks and caregiver community groups on WeChat, X (formerly Twitter), and Facebook, Participants were provided with an introductory statement explaining study objectives, ethical approval, and voluntary participation guidelines before beginning the survey. For the offline sample, paper questionnaires were distributed at partner hospitals and nursing institutions, with trained research assistants present to assist respondents if needed.

A total of 163 valid responses were collected. Among these, 89 respondents (54.6%) were recruited through hospitals, while 74 respondents (45.4%) were recruited through nursing institutions, With respect to recruitment channel, 94 participants (57.7%) were recruited online while 69 participants (42.3%) were recruited offline, This distribution ensured representation of both hospital-based and institution-based caregiving contexts, while leveraging online platforms to reach younger and digitally active caregivers.

Given the methodological requirements for structural equation modeling (SEM), psychological avoidance tendencies among caregiver populations, and the necessity for stable parameter estimates and adequate statistical power, the study aimed for at least 150 valid questionnaires. In May 2024, questionnaires were distributed throughout the Greater Bay Area. After eliminating incomplete or severely flawed responses, 163 valid samples remained for quantitative analysis. To enhance questionnaire design and clarity, the research team conducted brief supplementary interactions after questionnaire completion in select cases, involving short (1–2 min) follow-up questions such as “Which questions did you find difficult?” or “Did any options seem inconsistent with your actual experiences?” These interactions were neither audio-recorded nor thematically analyzed but rather served to verify item clarity and rationality for future questionnaire refinements; they were excluded from formal data analysis (Table 2).

**Table 2:** Demographic data.

Demographic	Type	Frequency	Percentage
Gender	Male	90	55.21%
	Female	73	44.79%
Age	18–25	98	60.12%
	26–35	27	16.56%
	36–45	17	10.43%
	Over 45	21	12.88%
Region	Central and southern Guangdong Province (Nine cities in the mainland)	97	59.51%
	East of the Pearl River Estuary (Hong Kong)	34	20.83%
	West of the Pearl River Estuary (Macao)	32	19.63%

**Figure 4:**
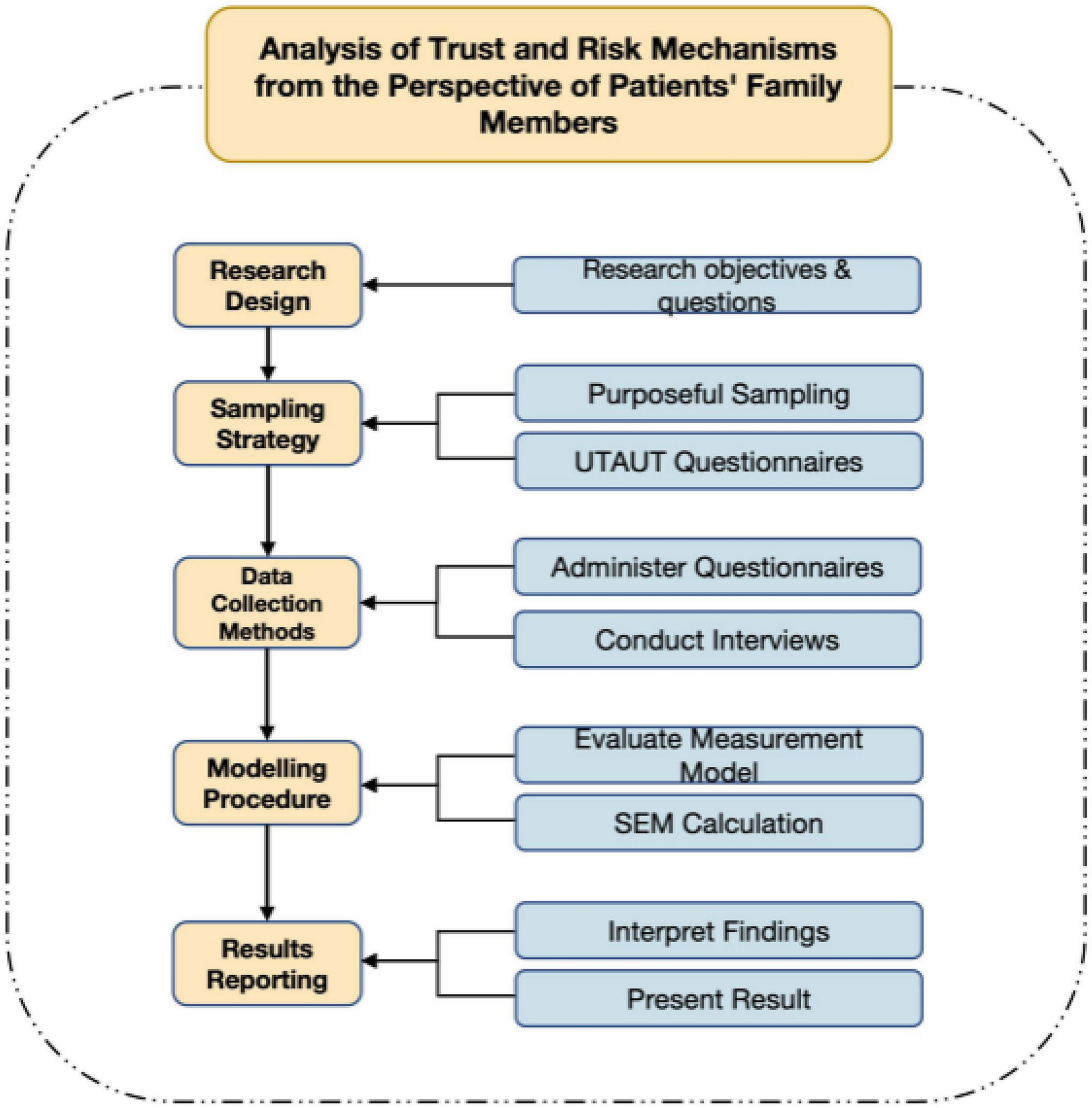
Research process.

For data analysis, this study utilized Partial Least Squares Structural Equation Modeling (PLS-SEM) via SmartPLS 4 software. PLS-SEM is well-suited for exploratory research involving complex models and is comparatively flexible with respect to sample size and data distribution assumptions. The analysis proceeded in two stages. First, the measurement model was evaluated by examining the reliability and validity of the latent constructs—specifically, Cronbach’s alpha and Average Variance Extracted (AVE)—as well as assessing multicollinearity through Variance Inflation Factors (VIF). Following the guideline by Hair et al. (41), VIF values exceeding 5 suggest problematic multicollinearity; in this study, all indicators showed VIF values well below this threshold, indicating no significant multicollinearity issues. Second, the structural model was assessed in terms of path coefficient significance, explanatory power (R^2^), and predictive relevance (Stone-Geisser’s Q^2^). Path coefficients were tested for statistical significance using a bootstrapping procedure with 5,000 resamples (Figure 4).

## 6 Research results

### 6.1 Reliability and validity assessment

This study used the Partial Least Squares (PLS) algorithm within SmartPLS 27 software to systematically examine constructs in the questionnaire, focusing primarily on key indicators such as Cronbach’s alpha, composite reliability (CR), and factor loadings.

Validity was evaluated through convergent validity and discriminant validity assessments. Convergent validity was assessed using Average Variance Extracted (AVE). As indicated in Table 3, all variables demonstrated AVE values exceeding the threshold of 0.5. Cronbach’s alpha values between 0.6 and 0.7 typically represent acceptable reliability (42). Behavioral Intention had the highest AVE (0.902). Discriminant validity was assessed based on the Fornell-Larcker criterion by comparing the square roots of AVE values with the correlation coefficients between variables (Table 4). All latent variables met discriminant validity requirements, consistent with Hair et al.’s recommendations for systematic PLS modeling (43).

**Table 3:** Results of internal consistency, or reliability, and concurrent validity testing.

Constructs	Items	Loadings	Cronbach’s alpha	Composite reliability (rho_c)	Average variance extracted (AVE)
PE	PE1	0.954	0.940	0.961	0.892
	PE2	0.952			
	PE3	0.928			
EE	EE1	0.906	0.819	0.892	0.735
	EE2	0.728			
	EE3	0.924			
SI	SI1	0.909	0.886	0.929	0.814
	SI2	0.918			
	SI3	0.879			
FC	FC1	0.915	0.899	0.937	0.832
	FC2	0.909			
	FC3	0.912			
Trust	Trust1	0.927	0.900	0.938	0.834
	Trust2	0.903			
	Trust3	0.909			
BI	BI1	0.948	0.946	0.965	0.902
	BI2	0.943			
	BI3	0.959			
UB	UB1	0.938	0.956	0.966	0.852
	UB2	0.927			
	UB3	0.889			
	UB4	0.891			
	UB5	0.966			

**Table 4:** Discriminant validity Fornell-Larcker test.

	Age	AI trust	BI	EE	FC	GDR	PE	PR	SI
Age	1.000								
AI trust	−0.128	0.913							
BI	−0.193	0.798	0.950						
EE	−0.178	0.583	0.610	0.857					
FC	−0.204	0.715	0.813	0.744	0.912				
GDR	0.120	0.180	0.163	0.176	0.241	1.000			
PE	−0.173	0.596	0.726	0.731	0.783	−0.107	0.945		
PR	−0.198	0.575	0.567	0.536	0.523	−0.097	0.483	1.000	
SI	−0.251	0.677	0.762	0.732	0.812	−0.118	0.773	0.545	0.902

Overall, the measurement model exhibited ideal levels of reliability, convergent validity, and discriminant validity. Empirical data showed that Cronbach’s alpha coefficients for all latent variables exceeded the acceptable threshold of 0.7, confirming excellent internal consistency and reliability (44). AVE values were all above 0.5, confirming convergent validity. Additionally, discriminant validity was confirmed by each construct’s square root of AVE being significantly higher than its correlations with other constructs, satisfying the Fornell-Larcker criterion (41).

### 6.2 Structural model assessment

The Variance Inflation Factor (VIF) was employed as the primary diagnostic tool for multicollinearity within PLS modeling. Hair et al. recommended that a VIF value above 5 indicates significant multicollinearity issues (Table 5).

**Table 5:** Multicollinearity statistics (VlF) for indicators.

Indicators	VIF
EE1	2.43
EE2	1.467
EE3	2.681
FC1	2.779
FC2	2.764
FC3	2.814
PE1	4.938
PE2	4.896
PE3	3.557
SI1	2.663
SI2	3.101
SI3	2.217
Trust1	3.27
Trust2	2.578
Trust3	2.796
Age	1
GDR	1
PR	1

All latent variables in this study had VIF values below 5, confirming the absence of serious multicollinearity and validating the questionnaire’s construct settings. This result further indicates that questionnaire items effectively distinguished among dimensions, thus minimizing biases and distortions from multicollinearity.

### 6.3 Model explanatory power assessment

In the Partial Least Squares Structural Equation Modeling (PLS-SEM) framework, researchers estimate path coefficients and factor loadings to maximize explained variance (R^2^) for endogenous latent variables (Table 6). The PLS method is particularly suitable for small sample sizes and complex models, offering effective predictions among latent constructs. Hair et al. established a standardized assessment framework classifying R^2^ effect sizes as strong (0.75), moderate (0.50), and weak (0.25).

**Table 6:** The explanatory power of the model R^2^.

	R-square	R-square adjusted
BI	0.832	0.814
UB	0.709	0.707

The results indicated a high explanatory power for behavioral intention (BI), with R^2^ = 0.832, significantly exceeding the typical UTAUT benchmark of 50–60%. This demonstrates that core predictors, such as performance expectancy and effort expectancy, explained 83% of the variance in behavioral intentions toward AI nursing applications. The adjusted R^2^ value (0.814), accounting for degrees of freedom, further underscored model robustness. Usage behavior (UB) had an R^2^ of 0.709 and an adjusted R^2^ of 0.707, showing that the model maintained strong explanatory power even after accounting for control variables and interaction effects.

### 6.4 Model fit assessment

Model fit was evaluated using the Standardized Root Mean Square Residual (SRMR). According to Henseler and Sarstedt (45), an SRMR value should be below 0.14. Analysis revealed that the SRMR value of the saturated model was 0.048, and the SRMR value of the estimated model was 0.069 (Table 7). Both values were significantly below the threshold, indicating acceptable model fit.

**Table 7:** Model fit indices comparison table.

	Saturated model	Estimated model
SRMR	0.048	0.069

### 6.5 Predictive relevance assessment

Predictive relevance (Q^2^) is a critical indicator of predictive validity in PLS models, ranging from −∞ to 1, with higher values indicating stronger predictive capability. Using the PLSpredict algorithm, predictive relevance for behavioral intention (BI) was Q^2^ = 0.762, and for usage behavior (UB) was Q^2^ = 0.727 (Table 8). Both values substantially exceeded 0, confirming the model’s strong predictive effectiveness.

**Table 8:** Predictive relevance assessment (Q^2^ predict).

	Q^2^ predict
BI	0.762
UB	0.727

### 6.6 Results analysis and discussion

**Figure 5:**
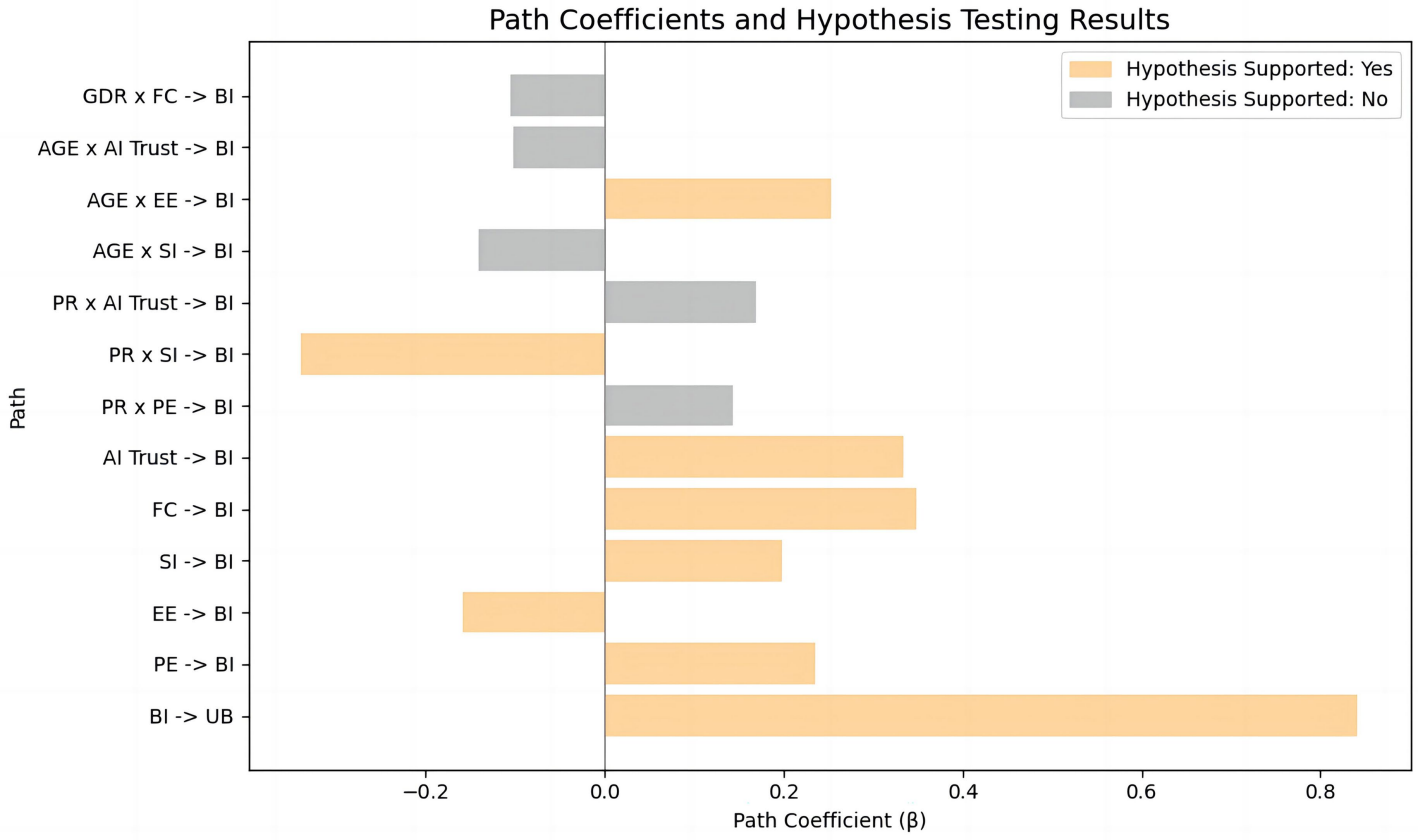
Path coefficients and hypothesis testing results.

**Figure 6:**
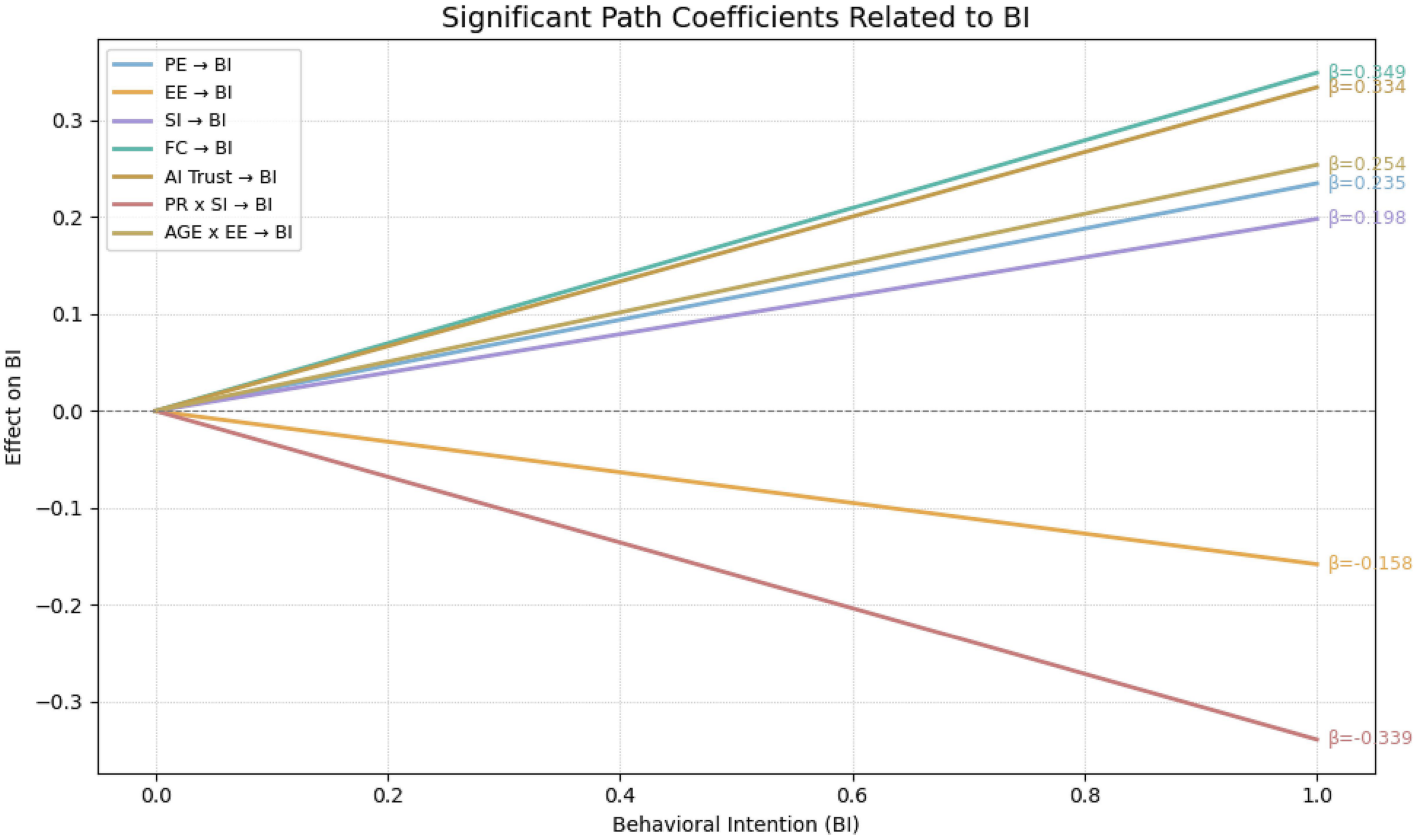
Dynamic path coefficients of behavioral intention (BI) determinants.

Bootstrapping with 5,000 resamples was utilized to calculate path coefficients and determine their significance. Path significance (T-statistic > 1.96 and *p*-value ideally < 0.05) determined hypothesis support, while the size of path coefficients indicated effect strength. Furthermore, the magnitude of each path coefficient reflects the relative strength of influence exerted by the independent variables on the corresponding dependent construct. The results of the hypothesis testing are summarized in Table 9. Figure 5 presents a bar chart illustrating the standardized β coefficients for each hypothesized path, with color coding used to differentiate between supported and unsupported hypotheses. Additionally, Figure 6 displays simple slope interaction plots, depicting variations in behavioral intention (BI) across different levels of key independent variables. These visualizations offer an intuitive representation of both the strength and directionality of the observed relationships.

**Table 9:** Path coefficients for hypothesis testing.

	Original sample (O)	Sample mean (M)	Standard deviation (STDEV)	T statistics (|O/STDEV|)	*p* values	Result
BI → UB	0.842	0.841	0.047	18.065	0.000	Supported
PE → BI	0.235	0.249	0.077	3.051	0.002	Supported
EE → BI	−0.158	−0.146	0.079	1.993	0.046	Supported
SI → BI	0.198	0.206	0.099	2.005	0.045	Supported
FC → BI	0.349	0.323	0.118	2.960	0.003	Supported
AI Trust → BI	0.334	0.329	0.084	3.964	0.000	Supported
PR × PE → BI	0.144	0.130	0.135	1.062	0.288	Not supported
PR × SI → BI	−0.339	−0.313	0.154	2.203	0.028	Supported
PR × AI Trust → BI	0.169	0.158	0.088	1.926	0.054	Not supported
AGE × SI → BI	−0.141	−0.132	0.099	1.417	0.157	Not supported
AGE × EE → BI	0.254	0.223	0.107	2.381	0.017	Supported
AGE × AI Trust → BI	−0.101	−0.082	0.084	1.196	0.232	Not supported
GDR × FC → BI	−0.105	−0.096	0.077	1.364	0.173	Not supported

#### 6.6.1 Supported hypotheses

The path from Behavioral Intention (BI) to Usage Behavior (UB) was highly significant (β = 0.842, t = 18.065, *p* < 0.001), confirming the strong translation from AI nursing intention to actual usage behavior. Performance Expectancy (PE) significantly enhanced BI (β = 0.235, t = 3.051, *p* = 0.002), supporting H1. Reverse-coded Effort Expectancy (EE) negatively influenced BI (β = −0.158, t = 1.993, *p* = 0.046), supporting H2. Social Influence (SI) positively affected BI (β = 0.198, t = 2.005, *p* = 0.045), supporting H3. Facilitating Conditions (FC) strongly drove BI (β = 0.349, t = 2.960, *p* = 0.003), supporting H4. AI Trust significantly predicted BI (β = 0.334, t = 3.964, *p* < 0.001), supporting H5. Age significantly amplified the negative influence of EE on BI (β = 0.254, t = 2.381, *p* = 0.017), supporting H7a. Perceived Risk (PR) negatively moderated the relationship between SI and BI (β = −0.339, t = 2.203, *p* = 0.028), supporting H6b.

#### 6.6.2 Unsupported hypotheses

Perceived Risk did not significantly moderate Performance Expectancy (H6a: β = 0.144, t = 1.062, *p* = 0.288), possibly due to institutional risk management in the Greater Bay Area (e.g., insurance, review mechanisms). The moderation effect of PR on AI Trust (H6c: β = 0.169, t = 1.926, *p* = 0.054) approached significance, suggesting potential clinical implications, possibly influenced by trust calibration mechanisms (46). Age did not significantly moderate the influence of SI on BI (H7b: β = −0.141, t = 1.417, *p* = 0.157), possibly due to older nurses prioritizing emotional goals over social evaluations. Similarly, Age did not significantly moderate AI Trust’s effect on BI (H7c: β = −0.101, t = 1.196, *p* = 0.232), perhaps reflecting heightened cognitive vigilance among older professionals in high-risk healthcare contexts. Gender’s moderating effect on FC (H8: β = −0.105, t = 1.364, *p* = 0.173) was not significant, likely reflecting diminished gender differences due to professional identity and widespread digital literacy.

## 7 Research significance and objectives

This study aimed to explore the factors influencing family caregivers’ acceptance of AI nursing technology and the underlying mechanisms, constructing and empirically testing a theoretical model integrating the Unified Theory of Acceptance and Use of Technology (UTAUT) with additional variables of trust and perceived risk. The study focused specifically on the Guangdong-Hong Kong-Macao Greater Bay Area—a region characterized by both international and local features—to provide valuable insights for promoting effective application of AI in nursing services within this region and beyond. The findings identified performance expectancy (β = 0.235, *p* < 0.01), facilitating conditions (β = 0.349, *p* < 0.01), and caregiver trust (β = 0.334, *p* < 0.001) as the three primary factors driving behavioral intention to use AI nursing. These results indicate that caregivers prioritize whether AI effectively reduces caregiving burdens (e.g., automated nursing reports), sufficient technical support from hospitals (e.g., 24-h equipment maintenance), and trust in algorithm reliability. Although social influence significantly enhanced usage intention (β = 0.198, *p* < 0.05), its effect was notably attenuated by perceived risk (moderation effect β = −0.339, *p* < 0.05). Furthermore, older caregivers exhibited significantly higher sensitivity to operational complexity (interaction effect of age and effort expectancy β = 0.254, *p* < 0.05), suggesting a need for simplified interaction designs. However, the moderating effect of age on social influence was not significant (*p* > 0.05), highlighting the rigorous and autonomous nature of caregivers’ decision-making processes regarding AI nursing.

Although AI systems in the Greater Bay Area span a wide spectrum—from monitoring sensors and scheduling tools to telehealth platforms and clinical decision support—their very heterogeneity contributes to uncertainty among caregivers. Even when certain systems demonstrate strong technical performance in pilot programs, caregivers often generalize concerns about malfunction, privacy, or operational complexity across all AI systems. This explains why our study found persistently low trust (β = 0.334, *p* < 0.001) despite evidence of effectiveness in similar contexts, and why perceived risk significantly weakened the effect of social influence (β = −0.339, *p* < 0.05). Building trust therefore requires not only improving algorithmic performance but also enhancing system transparency, caregiver training, and human-in-the-loop oversight mechanisms.

By focusing on caregivers as decision-makers and employing literature review and empirical analysis, this research advances social understanding of caregivers’ decision logic concerning AI nursing acceptance. It facilitates theoretical innovation in AI nursing research, shifting focus from technological efficacy to family acceptance mechanisms, and highlights how risk perception reshapes technology acceptance in safety-sensitive contexts.

Within the unique social-technological and healthcare context of the Guangdong-Hong Kong-Macao Greater Bay Area, this study holds particular research value. On the one hand, the region boasts advanced medical technologies and high digital literacy, potentially leading to overall greater acceptance of AI nursing. Conversely, the differences of institutional among cities (e.g., Hong Kong, Guangzhou, Macao) add valuable complexity to regional AI adoption research. This study aimed to identify regional variations in caregiver trust and risk perception and their effects on technology adoption intentions, thus informing region-specific policy development.

## 8 Research limitations and future directions

This study has several limitations. First, the sample size was relatively small. Our target population—primary family members actively involved in caregiving and medical decision-making—was inherently difficult to recruit, given substantial psychological and time pressures during patient care; strict adherence to ethical standards and informed consent without inducements further reduced response rates. Second, the sample was concentrated in the Guangdong–Hong Kong–Macao Greater Bay Area and skewed younger (ages 18–25 constituted 60.12%), which limits generalizability to other age groups and regions. Model- and design-related constraints should also be noted. Although the theoretical framework included trust and perceived risk, it did not fully capture deeper ethical constructs such as algorithmic transparency and accountability mechanisms, which may partly explain why certain moderation effects (e.g., H6a, H6c) were not significant. Moreover, the cross-sectional survey design cannot reflect dynamic shifts in technology acceptance—especially adaptations in caregiver trust following real-world AI exposure. Our ethical–legal discussion remains partly conceptual and jurisdiction-specific.

While the EU Artificial Intelligence Act (Regulation (EU) 2024/1689) establishes a risk-based regime, key implementation details and timelines are still evolving; by contrast, China currently regulates AI through sector-specific instruments rather than a single comprehensive act. Likewise, patient co-ownership models (e.g., GPOC) show early feasibility in the literature but have not yet been operationalized or evaluated in our context (47–49). To address these limitations, future work will:

- Pilot consent and portability pathways consistent with co-ownership principles;
- Conduct prospective fairness and harm audits (including subgroup performance and post-deployment monitoring) grounded in Ethics-by-Design and the four biomedical-ethics principles (50, 51);
- Align documentation (e.g., data-governance records, model cards, and logging) with emerging high-risk obligations and fundamental-rights protections highlighted by the EU framework.

In parallel, we will expand sample diversity—especially older decision-makers—strengthen cross-region comparisons, integrate ethical and institutional context variables, and adopt longitudinal designs to better illuminate trust-building mechanisms and risk-mitigation pathways in high-risk healthcare scenarios, thereby providing more precise decision-support for the sustainable implementation of smart nursing in aging societies.

Our findings also highlight the lack of formal training programs for caregivers in the use of AI-driven healthcare systems. Despite receiving some technical support, caregivers often struggled with system complexity and malfunction, which negatively affected their trust and willingness to adopt these technologies. Future studies should explore the development of structured, accessible training programs to enhance caregiver competency in using AI healthcare tools, especially for older caregivers who may face additional technological barriers. Moreover, it is essential that technical support be available, comprehensive, and responsive to caregivers’ needs to improve their experience with AI systems.

Finally, our study did not differentiate in detail between the multiple categories of AI-driven healthcare systems (e.g., monitoring, coordination, documentation), which may limit comparability across heterogeneous applications. Future research should stratify system types to better capture context-specific trust dynamics.

## Data Availability

The original contributions presented in the study are included in the article/supplementary material, further inquiries can be directed to the corresponding author.

## References

[1] Ministry of Civil Affairs and National Working Commission on Aging. (2024). 2023 annual report on the development of national undertakings for the elderly. The State Council of the People's Republic of China. Available online at: http://www.gov.cn/lianbo/bumen/202410/P020241012307602653540.pdf

[2] KPMG International. (2024). Realizing the value of AI in MedTech within Asia Pacific. Available online at: https://kpmg.com/cn/zh/home/insights/2025/03/realizing-the-value-of-ai-in-medtech-within-asia-pacific.html

[3] Caudevilla O. (2025). HK at forefront deploying AI in healthcare, innovation. China Daily Hong Kong. Available online at: https://www.chinadailyhk.com/hk/article/601492

[4] Lee AT Ramasamy RK Subbarao A. Understanding psychosocial barriers to healthcare technology adoption: a review of TAM technology acceptance model and unified theory of acceptance and use of technology and UTAUT frameworks. Healthcare. (2025) 13:250. doi: 10.3390/healthcare13030250, PMID: 39942440 PMC11816427

[5] Nong P Platt J. Patients’ trust in health systems to use artificial intelligence. JAMA Netw Open. (2025) 8:e2460628. doi: 10.1001/jamanetworkopen.2024.60628, PMID: 39951270 PMC11829222

[6] Park EH Werder K Cao L Ramesh B. Why do family members reject AI in health care? Competing effects of emotions. J Manag Inf Syst. (2022) 39:765–92. doi: 10.1080/07421222.2022.2096550

[7] Werder K Cao L Park EH Ramesh B. Why AI monitoring faces resistance and what healthcare organizations can do about it: an emotion-based perspective. J Med Internet Res. (2025) 27:e51785. doi: 10.2196/51785, PMID: 39889282 PMC11829173

[8] Kenesei Z Kökény L Ásványi K Jászberényi M. The central role of trust and perceived risk in the acceptance of autonomous vehicles in an integrated UTAUT model. Eur Transp Res Rev. (2025) 17:8. doi: 10.1186/s12544-024-00681-x

[9] Heidenreich S Kraemer T. Innovations—doomed to fail? Investigating strategies to overcome passive innovation resistance. J Prod Innov Manag. (2016) 33:277–97. doi: 10.1111/jpim.12273

[10] Kummer TF Recker J Bick M. Technology-induced anxiety: manifestations, cultural influences, and its effect on the adoption of sensor-based technology in German and Australian hospitals. Inf Manag. (2017) 54:73–89. doi: 10.1016/j.im.2016.04.002

[11] Kauttonen J Rousi R Alamaki A. Trust and acceptance challenges in the adoption of AI applications in health care: quantitative survey analysis. J Med Internet Res. (2025) 27:e65567. doi: 10.2196/65567, PMID: 40116853 PMC11971584

[12] Alaiad A Zhou L. The determinants of home healthcare robots adoption: an empirical investigation. Int J Med Inform. (2014) 83:825–40. doi: 10.1016/j.ijmedinf.2014.07.003, PMID: 25132284

[13] Gao Y Jiang Y Peng Y Yuan F Zhang X Wang J. Medical image segmentation: a comprehensive review of deep learning-based methods. Tomography. (2025) 11:52. doi: 10.3390/tomography11050052, PMID: 40423254 PMC12115501

[14] Martinez-Ortigosa A Martinez-Granados A Gil-Hernández E Rodriguez-Arrastia M Ropero-Padilla C Roman P. Applications of artificial intelligence in nursing care: a systematic review. J Nurs Manag. (2023) 2023:1–12. doi: 10.1155/2023/3219127, PMID: 40225652 PMC11919018

[15] Al Khatib I Ndiaye M. Examining the role of AI in changing the role of nurses in patient care: systematic review. JMIR Nurs. (2025) 8:e63335. doi: 10.2196/63335, PMID: 39970436 PMC11888071

[16] Topaz M Murga L Gaddis KM McDonald MV Bar-Bachar O Goldberg Y. Mining fall-related information in clinical notes: comparison of rule-based and novel word embedding-based machine learning approaches. J Biomed Inform. (2019) 90:103103. doi: 10.1016/j.jbi.2019.103103, PMID: 30639392

[17] Almagharbeh WT. The impact of AI-based decision support systems on nursing workflows in critical care units. Int Nurs Rev. (2025) 72:e13011. doi: 10.1111/inr.13011, PMID: 38973347

[18] Rony MKK Kayesh I Bala SD Akter F Parvin MR. Artificial intelligence in future nursing care: exploring perspectives of nursing professionals—a descriptive qualitative study. Heliyon. (2024) 10:e25718. doi: 10.1016/j.heliyon.2024.e25718, PMID: 38370178 PMC10869862

[19] Zhou LQ Wang JY Yu SY Wu GG Wei Q Deng YB. Artificial intelligence in medical imaging of the liver. World J Gastroenterol. (2019) 25:672–82. doi: 10.3748/wjg.v25.i6.672, PMID: 30783371 PMC6378542

[20] Robert N. How artificial intelligence is changing nursing. Nurs Manag. (2019) 50:30–9. doi: 10.1097/01.NUMA.0000578988.56622.21, PMID: 31425440 PMC7597764

[21] Loewenstein GF Weber EU Hsee CK Welch N. Risk as feelings. Psychol Bull. (2001) 127:267–86. doi: 10.1037/0033-2909.127.2.267, PMID: 11316014

[22] Buchanan C Howitt ML Wilson R Booth RG Risling T Bamford M. Predicted influences of artificial intelligence on the domains of nursing: scoping review. JMIR Nurs. (2020) 3:e23939. doi: 10.2196/23939, PMID: 34406963 PMC8373374

[23] Werder K Cao L Ramesh B Park EH. Empower diversity in AI development. Commun ACM. (2024) 67:31–4. doi: 10.1145/3676885

[24] Balagurunathan Y Mitchell R El Naqa I. Requirements and reliability of AI in the medical context. Phys Med. (2021) 83:72–8. doi: 10.1016/j.ejmp.2021.02.024, PMID: 33721700 PMC8915137

[25] China Radio International. (2025). Guangdong advances smart elderly care through technological innovation. Available online at: https://gd.cri.cn/n/20250318/4e7d6bc8-d694-3212-ca17-7b70b8887294.html

[26] Venkatesh V Morris MG. Why don't men ever stop to ask for directions? Gender, social influence, and their role in technology acceptance and usage behavior. MIS Q. (2000) 24:115–39. doi: 10.2307/3250981

[27] Kahnbach L Hase A Kuhl P Lehr D. Explaining primary school teachers’ intention to use digital learning platforms for students’ individualized practice: comparison of the standard UTAUT and an extended model. Front Educ. (2024) 9:1353020. doi: 10.3389/feduc.2024.1353020

[28] Hassan I Murad M El-Shekeil I Liu J. Extending the UTAUT2 model with a privacy calculus model to enhance the adoption of a health information application in Malaysia. Informatics. (2022) 9:31. doi: 10.3390/informatics9020031

[29] Fu K Ye C Wang Z Liu Z Wu M Yuan Y. Ethical dilemmas and the reconstruction of subjectivity in digital mourning in the age of AI: an empirical study on the acceptance intentions of bereaved family members of cancer patients. Front Digit Health. (2025) 7:1618169. doi: 10.3389/fdgth.2025.1618169, PMID: 40692654 PMC12278142

[30] Zeebaree M Agoyi M Aqel M. Sustainable adoption of e-government from the UTAUT perspective. Sustainability. (2022) 14:5370. doi: 10.3390/su14095370

[31] Rahi S Mansour M Alghizzawi M Alnaser F. Integration of UTAUT model in internet banking adoption context. J Res Interact Mark. (2019). doi: 10.1108/jrim-02-2018-0032

[32] Al-Saedi K Al-Emran M Ramayah T Abusham E. Developing a general extended UTAUT model for M-payment adoption. Technol Soc. (2020) 62:101293. doi: 10.1016/j.techsoc.2020.101293

[33] Lai CY Cheung KY Chan CS Law KK. Integrating the adapted UTAUT model with moral obligation, trust and perceived risk to predict ChatGPT adoption for assessment support: a survey with students. Comput Educ Artif Intelli. (2024) 6:100246. doi: 10.1016/j.caeai.2024.100246

[34] Cao J Kurata K Lim Y Sengoku S Kodama K. Social acceptance of mobile health among young adults in Japan: an extension of the UTAUT model. Int J Environ Res Public Health. (2022) 19:15156. doi: 10.3390/ijerph192215156, PMID: 36429875 PMC9690921

[35] Wennberg J Gittelsohn A. Small area variations in health care delivery: a population-based health information system can guide planning and regulatory decision-making. Science. (1973) 182:1102–8. doi: 10.1126/science.182.4117.1102, PMID: 4750608

[36] Kumar JA Bervell B. Google classroom for mobile learning in higher education: modelling the initial perceptions of students. Educ Inf Technol. (2019) 24:1793–817. doi: 10.1007/s10639-018-09858-z

[37] Hu S Laxman K Lee K. Exploring factors affecting academics’ adoption of emerging mobile technologies—an extended UTAUT perspective. Educ Inf Technol. (2020) 25:4615–35. doi: 10.1007/s10639-020-10171-x

[38] Glikson E Woolley AW. Human trust in artificial intelligence: review of empirical research. Acad Manag Ann. (2020) 14:627–60. doi: 10.5465/annals.2018.0057

[39] Morris M Schindehutte M Allen J. The entrepreneur's business model: toward a unified perspective. J Bus Res. (2005) 58:726–35. doi: 10.1016/j.jbusres.2003.11.001

[40] Carstensen LL Mikels JA Mather M. Aging and the intersection of cognition, motivation, and emotion In: Birren JE Schaie KW, editors. Handbook of the psychology of aging: Academic Press (2006). 343–62.

[41] Hair JF Sarstedt M Hopkins L Ringle CM. Partial least squares structural equation modeling: an emerging tool in business research. J Bus Res. (2018) 85:326–37.

[42] Ursachi G Horodnic IA Zait A. How reliable are measurement scales? External factors with indirect influence on reliability estimators. Proc Econ Finan. (2015) 20:679–86. doi: 10.1016/S2212-5671(15)00123-9

[43] Hair JF Sarstedt M Pieper TM Ringle CM. The use of partial least squares structural equation modeling in strategic management research: a review of past practices and recommendations for future applications. Long Range Plan. (2012) 45:320–40. doi: 10.1016/j.lrp.2012.09.008, PMID: 40955376

[44] Mator TA. Understanding Cronbach’s alpha: a guide to internal consistency reliability. J Med Educ. (2019) 84:1234–45.

[45] Henseler J Sarstedt M. Goodness-of-fit indices for partial least squares path modeling. Comput Stat. (2013) 28:565–80. doi: 10.1007/s00180-012-0317-1

[46] Zhang Y. Liao Q. V. Bellamy R. K. (2020). “Effect of confidence and explanation on accuracy and trust calibration in AI-assisted decision making.” in *Proceedings of the 2020 conference on fairness, accountability, and transparency*. pp. 295–305.

[47] Davids J ElSharkawy M Lidströmer N Ashrafian H Herlenius E. Technical sandbox for a global patient co-owned cloud (GPOC). BMC Digit Health. (2024) 2:74. doi: 10.1186/s44247-024-00128-2 PMC10928077 38467643

[48] Lidströmer N Davids J ElSharkawy M Ashrafian H Herlenius E. Systematic review and meta-analysis for a global patient co-owned cloud (GPOC). Nat Commun. (2024) 15:2186. doi: 10.1038/s41467-024-46503-5, PMID: 38467643 PMC10928077

[49] Lidströmer N Davids J ElSharkawy M Ashrafian H Herlenius E. A summit on a global patient co-owned cloud (GPOC). BMC Digital Health. (2024) 2:75. doi: 10.1186/s44247-024-00112-w, PMID: 38467643 PMC10928077

[50] Brey P Dainow B. Ethics by design for artificial intelligence. AI Ethics. (2024) 4:1265–77. doi: 10.1007/s43681-023-00330-4

[51] Knoppers BM Thorogood AM. Ethics and big data in health. Curr Opin Syst Biol. (2017) 4:53–7. doi: 10.1016/j.coisb.2017.07.001

